# Awake Craniotomy Program Implementation

**DOI:** 10.1001/jamanetworkopen.2023.52917

**Published:** 2024-01-24

**Authors:** Diogo Moniz-Garcia, Elird Bojaxhi, Bijan J. Borah, Ruchita Dholakia, Han Kim, Bernardo Sousa-Pinto, Joao Paulo Almeida, Marvesh Mendhi, William D. Freeman, Wendy Sherman, Lynda Christel, Steven Rosenfeld, Sanjeet S. Grewal, Erik H. Middlebrooks, David Sabsevitz, Benjamin F. Gruenbaum, Kaisorn L. Chaichana, Alfredo Quiñones-Hinojosa

**Affiliations:** 1 Department of Neurosurgery, Mayo Clinic Florida, Jacksonville; 2 Department of Anesthesiology, Mayo Clinic Florida, Jacksonville; 3 Robert D. and Patricia E. Kern Center for the Science of Health Care Delivery, Mayo Clinic, Rochester, Minnesota; 4 Division of Health Care Delivery Research, Department of Health Sciences Research, Mayo Clinic, Rochester, Minnesota; 5 Department of Community Medicine, Information and Health Decision Sciences, Faculty of Medicine, University of Porto, Porto, Portugal; 6 Center for Health Technology and Services Research, University of Porto, Porto, Portugal; 7 Department of Neurology, Mayo Clinic Florida, Jacksonville; 8 Department of Radiology, Mayo Clinic Florida, Jacksonville; 9 Department of Neuropsychology, Mayo Clinic Florida, Jacksonville

## Abstract

**Importance:**

Implementing multidisciplinary teams for treatment of complex brain tumors needing awake craniotomies is associated with significant costs. To date, there is a paucity of analysis on the cost utility of introducing advanced multidisciplinary standardized teams to enable awake craniotomies.

**Objective:**

To assess the cost utility of introducing a standardized program of awake craniotomies.

**Design, Setting, and Participants:**

A retrospective economic evaluation was conducted at Mayo Clinic Florida. All patients with single, unilateral lesions who underwent elective awake craniotomies between January 2016 and December 2021 were considered eligible for inclusion. The economic perspective of the health care institution and a time horizon of 1 year were considered. Data were analyzed from October 2022 to May 2023.

**Exposure:**

Treatment with an awake craniotomy before standardization (2016-2018) compared with treatment with awake craniotomy after standardization (2018-2021).

**Main Outcomes and Measures:**

Patient demographics, perioperative, and postoperative outcomes, including length of stay, intensive care (ICU) admission, extent of resection, readmission rates, and 1-year mortality were compared between patients undergoing surgery before and after standardization. Direct medical costs were estimated from Medicare reimbursement rates for all billed procedures. A cost-utility analysis was performed considering differences in direct medical costs and in 1-year mortality within the periods before and after standardization of procedures. Uncertainty was explored in probability sensitivity analysis.

**Results:**

A total of 164 patients (mean [SD] age, 49.9 [15.7] years; 98 [60%] male patients) were included in the study. Of those, 56 underwent surgery before and 108 after implementation of procedure standardization. Procedure standardization was associated with reductions in length of stay from a mean (SD) of 3.34 (1.79) to 2.46 (1.61) days (difference, 0.88 days; 95% CI, 0.33-1.42 days; *P* = .002), length of stay in ICU from a mean (SD) of 1.32 (0.69) to 0.99 (0.90) nights (difference, 0.33 nights; 95% CI, 0.06-0.60 nights; *P* = .02), 30-day readmission rate from 14% (8 patients) in the prestandardization cohort to 5% (5 patients) (difference, 9%; 95% CI, 19.6%-0.3%; *P* = .03), while extent of resection and intraoperative complication rates were similar between both cohorts. The standardized protocol was associated with mean (SD) savings of $7088.80 ($12 389.50) and decreases in 1-year mortality (dominant intervention). This protocol was found to be cost saving in 75.5% of all simulations in probability sensitivity analysis.

**Conclusions and Relevance:**

In this economic evaluation of standardization of awake craniotomy, there was a generalized reduction in length of stay, ICU admission time, and direct medical costs with implementation of an optimized protocol. This was achieved without compromising patient outcomes and with similar extent of resection, complication rates, and reduced readmission rates.

## Key Points

**Question:**

What is the cost utility of implementing standardized awake craniotomy programs for patients with brain tumors?

**Findings:**

In this economic evaluation including 164 patients, the standardized protocol was associated with mean (SD) savings of $7088.80 ($12 389.50) and decreases in 1-year mortality (dominant intervention). The standardized protocol was found to be cost saving in 75.5% of all simulations in probability sensitivity analysis.

**Meaning:**

Results of this study suggest that implementation of dedicated awake craniotomy teams is associated with cost savings and noninferior clinical outcomes.

## Teaser

This economic evaluation assesses the cost utility of introducing a standardized program of awake craniotomies.

## Introduction

The evolution of neuro-oncological surgery has led to progressively reduced surgical morbidity and enhanced patient recovery as new technologies and protocols are implemented.^1,2,3,4,5,6,7^ In fact, updated neuroanesthesia protocols, improved neuromonitoring and neuronavigation, and implementation of new surgical adjuncts have resulted in a reduced length of stay (LOS) and, in some cases, same-day discharge.^8,9,10,11,12^ Reduced LOS lowers exposure to potential nosocomial infections and thromboembolic complications, alleviates the psychological impact of brain tumor surgery, and is associated with higher patient satisfaction.^10,13,14^ Same-day discharge for neurosurgical cases was initially reported on minimally invasive procedures or biopsies; however, more recent studies have described significant reductions in inpatient admission rates for patients undergoing more complex surgical procedures such as supratentorial tumor resections^8^ and surgical clipping of intact cerebral aneurysms.^14^

Awake craniotomy represents a promising strategy that not only can reduce LOS but also may be a cost-effective treatment option. In fact, for appropriately selected patients, it has significant advantages over general anesthesia as it allows intraoperative stimulation for mapping of cortical function, dynamic functional monitoring of neurological status, and decreased hemodynamic fluctuation.^15,16,17,18,19^ However, limited literature exists on the overall impact and benefit to hospitals in investing in these subspecialized and resource-intensive programs. We hypothesized that for awake craniotomies, the introduction of standardized practices in perioperative anesthetic treatment would lead to expedited patient turnover with reduced LOS and admission time at the intensive care unit (ICU), while ensuring similar or better patient outcomes and potentially generating cost savings. Therefore, in this study, we aim to provide a full economic evaluation analysis (cost-utility study) of awake craniotomy in the context of dedicated teams and adequate patient selection, assessing whether there were gains in hospital efficiency while maintaining quality patient outcomes.

## Methods

We performed a single-institution, retrospective cohort study associated with a cost-utility study to evaluate the outcomes of the standardization in our institution’s awake craniotomy program. Data on costs and health services use were estimated adopting the health care institution perspective, considering direct medical costs associated with the surgical procedure and with the corresponding inpatient admission. A time horizon of 1 year was considered. This study was reviewed and approved by the Mayo Clinic institutional review board (IRB). Patient consent was not needed given the retrospective nature of the study, as per the IRB. Consolidated Health Economic Evaluation Reporting Standards (CHEERS) reporting guidelines were followed.

### Patient Selection, Data Collection, and Management

We included all adult patients with single, unilateral lesions who underwent elective awake craniotomies between January 2016 and December 2021 (excluding preadmitted patients) at Mayo Clinic Florida. Patients with emergent cases, preoperative opioid use, chronic pain history, and major comorbidities as highlighted by the American Society of Anesthesiologists Physical Status Classification IV were excluded. The outcomes of standardization were assessed by comparing patients that had the surgery in the prestandardization period (2016–2018) with patients in the poststandardization pereiod (2018–2021).

Patient demographic and clinical data were collected from patients’ electronic medical records. Surgical, imaging, and pathology data were also assessed. Volumetric measurements were obtained manually, defining the region of interest on each scan of each slide in the axial plane, with subsequent computerization of the volumetric data with Digital Imaging and Communications in Medicine medical image viewer software OsiriX (Pixmeo SARL). Early postoperative characteristics, including LOS, ICU admission time, postoperative seizures, and readmission within 30 days were also evaluated. Finally, we evaluated mortality rates at 1 year after surgery.

In addition to the primary cost-utility assessment involving all patients, a subset cost-utility analysis was also conducted restricted to only patients with glioma. Propensity score matching was performed using SPSS Statistics, version 22 (IBM), which sought to balance age, sex, World Health Organization (WHO) grade, and isocitrate dehydrogenase (IDH) variation status between the intervention and control patients.

### Prestandardized Protocol

Patients underwent a presurgical assessment with the neurosurgeon, where they received initial orientation. The neuroanesthesiologist provided further details on the perioperative course using the asleep/awake/asleep technique (AAA) on the morning of the surgery. Before the procedure, the patient visited the preoperative evaluation clinic, where a member of our internal medicine team focused on cardiopulmonary assessment and medical optimization. The AAA technique involved the following steps: induction of general anesthesia (GA) with propofol, insertion of a laryngeal mask airway (LMA), and maintenance of anesthesia with a combination of propofol infusion and halogenated gases. During GA, the patient’s head was immobilized using pins, a scalp block was performed using the landmark technique, and surgical exposure was achieved. Once the dura was exposed, the patient emerged from GA, the LMA was removed, and neurocognitive testing was initiated. For surgical closure, GA was reinduced, and an LMA was reinserted. Following the procedure, all patients were transferred to the ICU for postoperative management. However, goals for advancing the diet, ambulation, and removal of catheters were not standardized. Opioids were the primary strategy for postoperative pain management.

### Awake Craniotomy Multidisciplinary Standardized Protocol

Standardization involved a dedicated awake craniotomy team involving neurosurgery, neuroanesthesiology, neuropsychology, and dedicated nursing and operating room (OR) teams with standardization of protocols throughout. Every patient had a preoperative neuropsychology visit for baseline evaluation and optimization of surgical planning. All patients had a preoperative evaluation with an anesthesiologist. Detailed instructions of the perioperative course were discussed and educational material provided. To reduce the risk of postoperative nausea and the use of intravenous opioids for intractable pain, a multimodal regimen was started preoperatively. In the holding area, the patient received oral celecoxib, acetaminophen, and aprepitant. The patient was given routine doses of acetaminophen every 6 hours with a maximum dose of 4 g per day. Clear liquids were started on postoperative day (POD) 0 and the patient advanced to a regular diet by POD 1. Oral analgesics were encouraged over intravenous administration. The standardization of the perioperative anesthetic course moved away from an AAA technique, and the focus was placed on an ultrasound-guided regional anesthetic technique and intermittent conscious or deep sedation. In the preoperative area, a selective ultrasound-guided scalp block was performed under minimal sedation. A dexmedetomidine infusion was titrated for the block while maintaining a meaningful verbal interaction with the patient. Ultrasound-guided bilateral greater occipital and bilateral supraorbital nerve blocks were performed in anticipation of the headframe pin locations. The lesser occipital and auriculotemporal nerves were blocked ipsilateral to the surgical side only. To address discomfort seen with dissection of the temporalis muscle off the temporal bone,^7^ we performed an ipsilateral deep temporal nerve block under direct ultrasound guidance. Approximately 20 ml of 0.5% ropivacaine with 1:200 000 of epinephrine was injected in total. A weight-based maximum local anesthetic dose was calculated and communicated to the surgical team. Before transferring to the OR, decreased sensation over the anticipated surgical location was confirmed, and the patient’s cognitive and neurological status returned to baseline. Upon arrival to the OR, conscious to deep sedation was initiated and included an infusion of dexmedetomidine and low dose propofol (approximately 20 µg/kg/min) as needed. During surgical time-out, compliance with the protocol was assessed with the neuropsychology team, neuroanesthesiology team, and neurosurgery team, as well as nursing and OR dedicated staff. During surgical exposure, analgesia for breakthrough pain was addressed with additional local anesthetic (2% lidocaine with 1:200 000 epinephrine) injected by the surgeon, or intravenous fentanyl. All sedative medications were discontinued once the bone flap was removed. The neuropsychologist was present during the entire perioperative course to provide feedback and verbal reassurance to the patient. Conscious to deep sedation was resumed during closure and titrated with the goal of reawakening the patient at the end of the surgery to reassess neurocognitive function before leaving the OR.

Enhanced recovery after surgery stipulations were also followed. The anticipated need for lines and drain were discussed with the operative team before the surgery. Arterial line placement was deferred if the patient was normotensive and did not have a history of vascular disease, and a urinary catheter was avoided for procedures less than 2 hours. Intraoperative diuretics were not typically used, to avoid excessive distortion of the brain anatomy and headaches due to intracranial hypotension. If a urinary catheter was placed, it was discontinued on POD 0 as the patient was encouraged to transfer to a chair and use the bedside commode. In the ICU, the patient was monitored every hour for acute neurocognitive changes over 24 hours. On POD 1, a magnetic resonance imaging exam was performed, and the patient was downgraded to the postsurgical ward if no complications were apparent. Discharge plans were typically made by POD 2 assuming the patient was tolerating a regular diet and oral medications and was able to participate in their own care. Postdischarge follow-up information was provided.

### Cost Data

We assessed direct medical costs, including costs associated with the OR, anesthesia, other health care clinicians, board, room, and miscellaneous costs (such as imaging, laboratory tests, and drugs). All costs used in the study were obtained from Mayo Clinic’s standardized cost data warehouse.^5,20,21^ Broadly speaking, the method assigns Medicare reimbursement rates to all professionally billed services for a specific year. For hospital services, the Medicare cost-to-charge ratios were multiplied by the associated charges to create standardized costs. All the costs obtained by both methods were converted to 2018 US dollars using the gross domestic product implicit price deflator.

### Statistical Analysis

Categorical variables were described using absolute and relative frequencies, while continuous variables were described using means and SDs or medians and IQRs. Patients undergoing surgery before and after standardization were compared in premorbid indicators, perioperative outcomes, and postoperative outcomes. Costs associated with both cohorts were compared via simple *t* test.

To perform a complete economic evaluation, incremental costs (ie, differences in costs obtained with vs without procedure standardization) were calculated and assessed in relation to differences in 1-year mortality. An incremental cost-utility ratio of less than 50 000 US dollars per life year gained was considered indicative that poststandardization craniotomy was cost-effective. To assess the robustness of our results, we performed probabilistic sensitivity analysis via Monte Carlo simulation methods^22^ using 10 000 simulations with variables not assuming base case input values, but rather randomly drawn from their corresponding probability distributions. Gamma distributions were defined for cost variables (defined based on mean [SD] values obtained for those values in our samples), while β distributions were defined for 1-year mortality (defined based on the probabilities observed in our sample). Probabilistic sensitivity analysis was performed using R software version 4.0 (R Project for Statistical Computing). A deterministic sensitivity analysis was also performed. For each cost-category, a variation of 1 SD was considered and the most cost-effective strategy evaluated. All tests were 2-sided and a *P* value of .05 was considered significant. Data were analyzed from October 2022 to May 2023.

## Results

A total of 164 patients (mean [SD] age, 49.9 [15.7] years; 98 [60%] male patients) were included in this study, including 56 patients prestandardization and 108 poststandardization (Video). Health care use and cost data were retrieved following the evaluation of all patients in the study (eFigure 1 in Supplement 1). Patients in both groups presented with similar distribution of age, sex, body mass index, pathological diagnosis, and preoperative tumor volume (Table 1).

**Video.** 3-Dimensional Reconstruction of Dominant Hemisphere Gliomas A, A 3-dimensional reconstruction of diffusion tensor imaging of a man aged 41 years with dominant hemisphere glioma demonstrating the tumor and its relationships to the adjacent inferior fronto-occipital fasciculus and uncinate fasciculus. B, A 3-dimensional reconstruction of diffusion tensor imaging of a man aged 30 years with dominant hemisphere glioma demonstrating the close relationship between the infiltrating tumor and the adjacent inferior longitudinal fasciculus, corticospinal tracts, and frontal aslant tract. Image of man aged 30 years with dominant hemisphere glioma demonstrating the close relationship between the infiltrating tumor and the adjacent inferior longitudinal fasciculus, cortico-spinal tracts, frontal aslant tract.

**Table 1.** Preoperative Characteristics and Perioperative Patient Outcomes for Patients Undergoing Awake Craniotomy Before and After Implementation of the Multidisciplinary Standardization Program

Patient characteristics	Patients, No. (%)			*P* value
	All patients (N = 164)	Prestandardization (n = 56)	Poststandardization (n = 108)	
Age at surgery, median (IQR), y	49.0 (36.5-63.0)	49.0 (36.5-63.0)	49.5 (36.5-63.0)	.95
Sex				
Male	98 (60)	34 (61)	64 (59)	.87
Female	66 (40)	22 (39)	44 (41)	
No. of comorbidities, median (IQR)	1 (0-3)	1 (0-2)	1 (0-3)	.23
Body mass index, median (IQR)^a^	27.3 (23.6-27.3)	26.6 (22.1-29.8)	27.7 (23.5-30.7)	.10
Obesity	42 (26)	14 (25)	28 (26)	.99
Hypertension	48 (29)	21 (38)	27 (25)	.11
Diabetes	13 (8)	5 (9)	8 (7)	.77
Hyperlipidaemia	39 (24)	14 (25)	25 (23)	.85
Coronary artery disease	3 (2)	1 (2)	2 (2)	.99
Heart failure	1 (1)	0	1 (1)	.99
Chronic kidney disease	8 (5)	3 (5)	5 (5)	.99
Stroke	1 (1)	0	1 (1)	.99
Arrythmia	8 (5)	1 (2)	7 (6)	.26
DVT	1 (1)	0	1 (1)	.99
Asthma	10 (6)	3 (5)	7 (6)	.99
OSA	5 (3)	1 (2)	4 (4)	.66
Preoperative KPS	83	83	81	.42
Intraoperative complications	2 (1)	2(4)^b^	0	.12
Volumetric extent of resection, %	83.07	83.79	82.74	.32
ICU admission, median, nights	1	1	1	.02
LOS, median, nights	2	3	2	.002
Readmission 30 d	13 (8)	8 (14)	5 (5)	.03
One-year mortality	25 (16)	9 (16)^c^	16 (16)^d^	.03

Abbreviations: DVT, deep venous thrombosis; ICU, intensive care unit; KPS, Karnofsky Performance Scale; LOS, length of stay; OSA, obstructive sleep apnea.

^a^ Body mass index is calculated as weight in kilograms divided by height in meters squared.

^b^ Left upper extremity weakness and perioperative ischemic infarction.

^c^ One patient lost to follow-up.

^d^ Six patients lost to follow-up.

### Perioperative and Postoperative Outcomes

Perioperative and postoperative outcomes for both groups are summarized in Table 1. OR time was reduced from a mean (SD) of 201 (60.7) minutes prestandardization to a mean (SD) of 165.3 (34.9) minutes poststandardization (difference, 35.7 minutes; 95% CI, 20.9-50.5 minutes; *P* = .01). In the prestandardization cohort, all patients required ICU admission. Standardization was associated with a significant reduction in LOS, from a mean (SD) of 3.34 (1.79) days prestandardization to a mean (SD) of 2.46 (1.61) poststandardization (difference, 0.88 days; 95% CI, 0.33-1.42 days; *P* = .02), with 19% (20 of 108) of patients bypassing ICU admission and 12% (13 of 108) having same-day discharge. Mean (SD) admission time at the ICU was reduced from a mean (SD) of 1.32 (0.69) to 0.99 (0.90) nights (difference, 0.33 nights; 95% CI, 0.06-0.60 nights; *P* = .02). Patients undergoing awake poststandardization surgery had significantly lower rates in 30-day readmission, from 14% (8 patients) in the prestandardization cohort to 5% (5 patients) poststandardization (*P* = .03). A total of 7 patients were lost to follow-up at 1-year time point.

### Cost Data

Costs for both groups are summarized in Table 2 and eFigure 2 in Supplement 1. The total costs for the poststandardization cohort (mean: $28 919; 95% CI, $26 476-$31 361) were found to be significantly lower than those of the prestandardization cohort ($35 981; 95% CI, $32 630-$39 931; *P* < .001), with significant reductions in total OR costs (poststandardization mean, $10 445; 95% CI, $10 051-$10 839; prestandardization mean, $13 383; 95% CI, $12 970-$13 795; *P* < .001); costs associated with health care clinicians, including surgeons, nurses, and surgical technicians (poststandardization mean, $5608; 95% CI, $5306-$5911; prestandardization mean, $10 934; 95% CI, $10 159-$11 708; *P* < .001); and room and board costs (poststandardization mean, $3805; 95% CI, $2912-$4698; prestandardization mean, $6792; 95% CI, $5456-$8159; *P* < .001). Total anesthesia costs were found to be similar between cohorts (poststandardization mean, $1320; 95% CI, $1224-$1416; prestandardization mean, $1312; 95% CI, $1168-$1457; *P* = .93). Costs associated with laboratory tests, imaging, and medication were found to be higher in the poststandardization cohort (poststandardization mean, $7777; 95% CI, $6340-$9213; prestandardization mean, $3560; 95% CI, $1412-$5708; *P* < .001). Costs for the subanalysis, restricted to glioma patients, matched by age, gender, WHO grade, and IDH variation status, are summarized in Table 3. Similar findings were observed with total costs, operating costs, costs associated with health care professionals, and room and board costs were found to be significantly reduced in the poststandardization period, but the results were not significantly different between anesthesia costs (post-standardization mean, $1360; 95% CI, $1192-$1528; pre-standardization mean, $1302; 95% CI, $1132-$1472; *P* = .63). Analysis of other intracranial procedures at our institution for the period in analysis did not show that these results were part of an existing trend; the mean (SD) LOS for elective arteriovenous malformations in the period before standardization was 4.82 (1.54) nights and after was 4.71 (1.43) nights.

**Table 2.** Costs for Entire Cohort Included in the Study

Inputs	Cost, US $^a^		*P* value
	Mean (SD)	95% CI	
Operating room and material costs			
Cohort prestandardization	13 383 (2471.69)	12 970-13 795	<.001
Cohort poststandardization	10 445 (1808.15)	10 051-10 839	
Health clinician costs			
Cohort prestandardization	10 934 (4461.37)	10 159-11 708	<.001
Cohort poststandardization	5608 (1203.71)	5306-5911	
Anesthesia costs			
Cohort prestandardization	1312 (600.60)	1168-1457	.93
Cohort poststandardization	1320 (441.53)	1224-1416	
Room costs			
Cohort prestandardization	6792 (4040.75)	5456-8159	<.001
Cohort poststandardization	3805 (4807.60)	2912-4698	
Other costs			
Cohort prestandardization	3560 (3640.45)	1412-5708	<.001
Cohort poststandardization	7777 (8504.55)	6340-9213	
Total costs			
Cohort prestandardization	35 981 (6830.79)	32 630-39 931	<.001
Cohort poststandardization	28 919 (13 023.52)	26 476-31 361	

^a^ Costs extracted from Mayo Clinic’s standardized cost warehouse using Medicare reimbursement rates.

**Table 3.** Costs for Subgroup Analysis Restricted to Glioma Patients With Matched Participants

Inputs	Cost, US $^a^		*P* value
	Mean (SD)	95% CI	
Operating room and material costs			
Cohort prestandardization	13 245 (2286.00)	12 633-13 857	<.001
Cohort poststandardization	10 730 (1600.94)	10 125-11 334	
Health clinician costs			
Cohort prestandardization	11 009 (4156.73)	10 086-11 932	<.001
Cohort poststandardization	5652 (753.21)	4741-6564	
Anesthesia costs			
Cohort prestandardization	1302 (638.11)	1132-1472	.63
Cohort poststandardization	1360 (4442.39)	1192-1528	
Room costs			
Cohort prestandardization	5835 (3603.55)	4953-6717	<.001
Cohort poststandardization	3492 (1805.90)	2620-4364	
Other costs			
Cohort prestandardization	3315 (3315.32)	1960-4670	<.001
Cohort poststandardization	7565 (7564.68)	6226-8904	
Total costs			
Cohort prestandardization	34 706 (6413.99)	32 604-36 810	<.001
Cohort poststandardization	28 799 (7096.64)	26 721-30 877	

^a^ Costs extracted from Mayo Clinic’s standardized cost warehouse using Medicare reimbursement rates.

### Cost-Utility Analysis

We observed lower costs and lower 1-year mortality associated with the standardized program. Therefore, the standardized program may be considered a dominant intervention, rendering the computation of incremental cost-utility ratios irrelevant. Results of probabilistic sensitivity analyses are displayed in Table 4. The standardized protocol was found to be cost saving in 75.5% of all simulations (Figure, A), associated with mean (SD) savings of $7088.80 ($12 389.50). Similar results were obtained for the subanalysis restricted to glioma patients with matched cohorts, where the standardized protocol was found to be cost saving in 75.9% of all simulations (Figure, B). Results of a deterministic sensitivity analysis demonstrated the cost-effectiveness of the standardized protocol in every scenario, except a worst-case scenario for other costs in the poststandardization cohort (eTable in Supplement 1).

**Table 4.** Results of the Probabilistic Sensitivity Analysis Comparing the Cohort Before and After Protocol Standardization

Probabilistic Analysis	Outputs, No. (%)
Difference in costs, mean (SD), $US	−7088.8 (12 389.5)
Difference in 1-y survival, mean (SD), %	0.6 (6.2)
Simulations in which the standardized protocol found to be cost-saving	7551 (75.5)
Subgroup analysis	
Difference in costs, mean (SD), $US	−5988.0 (8976.3)
Difference in 1-y survival, mean (SD), %	0.7 (6.1)
Simulations in which the standardized protocol found to be cost-saving	7593 (75.9)
Simulations in which the standardized protocol found to be associated with increased 1-y survival	5344 (53.4)
Simulations in which the standardized protocol found to be cost-saving and associated with increased 1y survival	4044 (40.4)
Simulations in which the standardized protocol found to be more expensive and associated with lower 1y survival	1105 (11.1)

**Figure.**
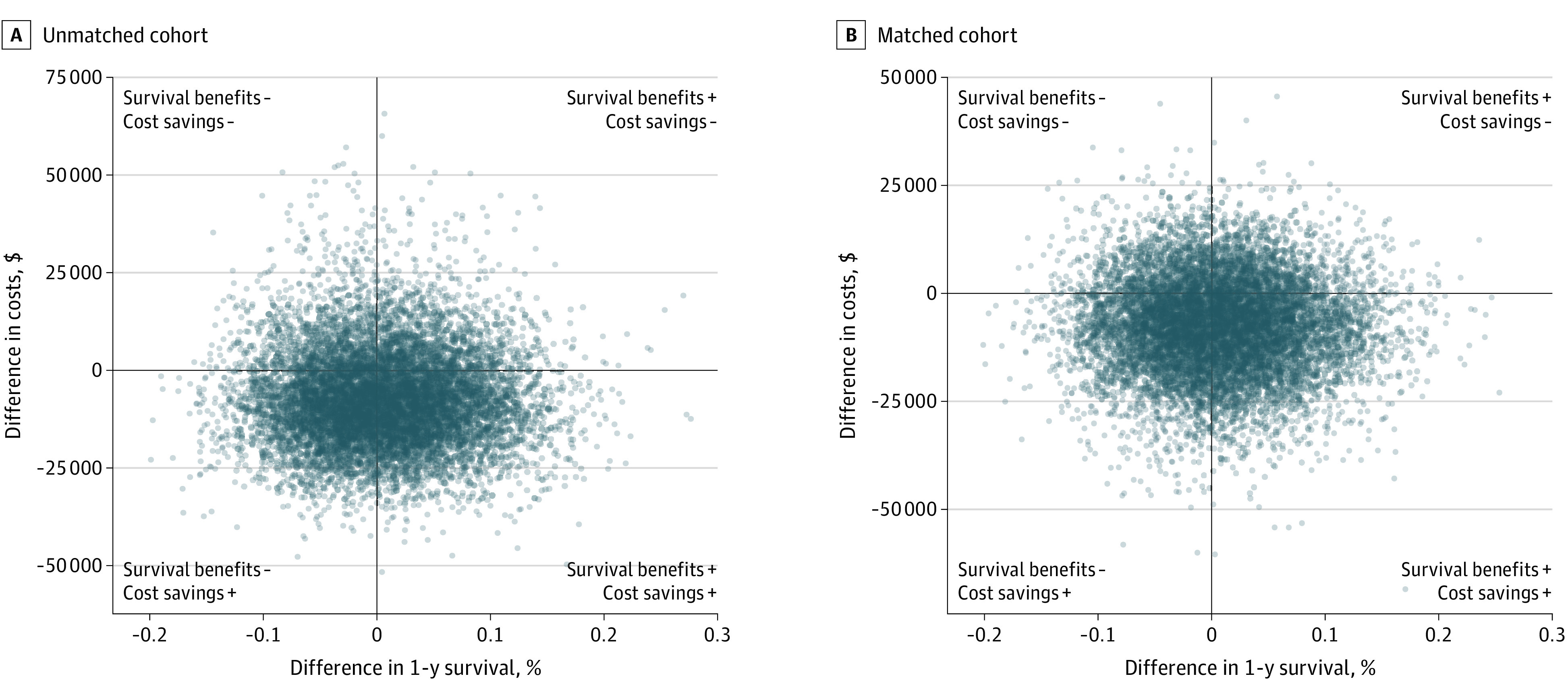
Scatterplot of Sensitivity Analysis Scatterplot illustrating the results of probabilistic sensitivity analysis comparing A, cohort before and after standardization of protocols, and B, subcohort, restricted to gliomas and matched before and after standardization. Each dot corresponds to the result of 1 simulation (ie, 1 comparison between both cohorts); the location of each dot in the vertical axis indicates the difference in costs between both cohorts for the respective simulation, while its location in the horizontal axis indicates the difference in 1-year survival.

## Discussion

In this study, we report our experience implementing a multidisciplinary awake tumor program (Video) with reduction in overall mean LOS, ICU usage, improvement in 30-day readmission rate, and reduction in overall costs by a mean of $7088.80 (resulting from reductions in costs of the OR, health care clinicians, and room and board). However, our results should be carefully interpreted as they are based on the data of a single institution.

As health care costs increase in developed nations, particularly in the US, there is an increasing focus on strategies that maintain or improve patient outcomes while being economically efficient.^5,23^ In neurosurgery, and brain tumor surgery in particular, multiple centers have reported their experience decreasing mean LOS and achieving same-day discharge.^8,9,10,11,12^ But progress to reduce mean LOS for awake craniotomy lags behind other surgical specialties. Concerns regarding short-term risk of deterioration after same-day discharge have prevented a wider adoption of standards and practices aimed at same-day craniotomies and faster discharge times. Regardless, multiple centers have attempted to change their practices to enable same-day discharge.^8,9,10^ Recently, Vallejo et al^8^ reported on the implementation of an outpatient craniotomy program applicable to 37 of 334 patients undergoing craniotomy, with successful same-day discharge in 32 patients. Importantly, that study included patients with supratentorial tumors of fairly small size for surgical procedures with low predicted estimated blood loss and operating time.

Our experience highlights important lessons learned from our awake craniotomy program that allowed for some patients to bypass the ICU with same-day discharge. Furthermore, we observed a decrease in LOS with mean total cost savings of $7088.80, translating into a total mean savings of $765 590.40 in prevented costs. Importantly, perioperative and postoperative outcomes, including extent of resection, new reported deficits, new seizures, 30-day readmission rates, and death, were not significantly increased with shorter LOS. The bypass of ICU for some patients freed up ICU beds for additional surgical procedures or patients in the ICU with higher overall acuity, which benefits the institution and these patients but was not accounted for in this analysis.

### Limitations and Strengths 

This study has important potential limitations. Our cost estimates were derived from the experience of a single institution, which could limit their external validity. However, we addressed this by using Medicare reimbursement rates, which should increase the applicability of our observations to other institutions. Furthermore, we reported a retrospective cohort, which could limit some of our conclusions. Moreover, information on utility data, as well as on indirect costs or out-of-pocket costs to patients, were not considered. This is because the retrospective design of our study precluded us from collecting quality of life or utility data and indirect costs from our patients. Importantly, this is a before-and-after study, so 2 different time periods were compared, which could suggest the role of confounding factors with effects on resource management and procedure time and costs. However, we did not observe significant differences in procedure costs for the period being analyzed, including in cost of supplies and supplier mix. Our results could reflect an overall existing trend toward shorter LOS. However, analysis of other intracranial procedures at our institution for the period in analysis did not confirm such an existing trend (for example, the mean LOS for elective arteriovenous malformations in the period before standardization was 4.82 nights, and after was 4.71 nights), with the same being observed for elective aneurysm clipping and bypass procedures. Furthermore, comparison of before-and-after costs (eFigure 2 in Supplement 1) demonstrate the outcomes of the intervention.

Overall, this study provides a useful guide to improve efficacy in awake craniotomies, improving the patient experience and the cost to the hospital. It is the first study we know of evaluating the outcomes of the standardization of protocols and practices and the optimization of a multidisciplinary team in awake craniotomy in both patient outcomes and economic results. This, in turn, will potentially open the discussion on the implementation of similar protocols in other institutions, given the demonstrable advantages to both patients and health care services. It should be noted that multiple factors affect the cost of awake tumor surgery, with considerations varying from institution to institution. However, the use of reimbursement rates in this report increases its external validity to institutions with differing cost structures. Our study also includes consecutively enrolled patients over a considerable time horizon with patients similar at baseline between comparison periods.

## Conclusions

In this economic evaluation of patients undergoing awake craniotomy, the standardization of procedures and the involvement of a multidisciplinary dedicated team resulted in an overall decrease in mean length of stay, ICU stay, and direct medical costs. These decreases occurred without overall worse perioperative patient outcomes or 1-year mortality.

## Notes

Supplement 1. **eFigure 1.** Illustrative Case of Man Aged 41 Years With Dominant Hemisphere Insular Glioma That Underwent Awake Craniotomy Surgery, Demonstrating the Multidisciplinary Approach Established in Study Program **eFigure 2.** Trend Plots Illustrating Cost Changes Over Time for Both Prestandardization and Poststandardization Cohorts **eTable.** Sensitivity Analysis Considering Variable Inputs up to 1 SD

Supplement 2.
Data Sharing Statement
