# Supplementary material for: Awake Craniotomy Program Implementation
Source: JAMA Netw Open. 2024 Jan 24;7(1):e2352917. doi: 10.1001/jamanetworkopen.2023.52917 (PMC10809012; doi:10.1001/jamanetworkopen.2023.52917)
Supplement: Supplement 1. — eFigure 1. Illustrative Case of Man Aged 41 Years With Dominant Hemisphere Insular Glioma That Underwent Awake Craniotomy Surgery, Demonstrating the Multidisciplinary Approach Established in Study Program eFigure 2. Trend Plots Illustrating Cost Changes Over Time for Both Prestandardization and Poststandardization Cohorts eTable. Sensitivity Analysis Considering Variable Inputs up to 1 SD [file jamanetwopen-e2352917-s001.pdf]

Supplemental Online Content

Moniz-Garcia D, Bojaxhi E, Borah BJ, et al. Awake craniotomy program implementation. *JAMA Netw Open*. 2023;7(1):e2352917. doi:10.1001/jamanetworkopen.2023.52917

eFigure 1. Illustrative Case of Man Aged 41 Years With Dominant Hemisphere Insular Glioma That Underwent Awake Craniotomy Surgery, Demonstrating the Multidisciplinary Approach Established in Study Program
eFigure 2. Trend Plots Illustrating Cost Changes Over Time for Both Prestandardization and Poststandardization Cohorts
eTable. Sensitivity Analysis Considering Variable Inputs up to 1 SD

This supplemental material has been provided by the authors to give readers additional information about their work.

© 2024 Moniz-Garcia D et al. *JAMA Network Open.*

eFigure 1. Illustrative Case of Man Aged 41 Years With Dominant Hemisphere Insular Glioma That Underwent Awake Craniotomy Surgery, Demonstrating the Multidisciplinary Approach Established in Study Program

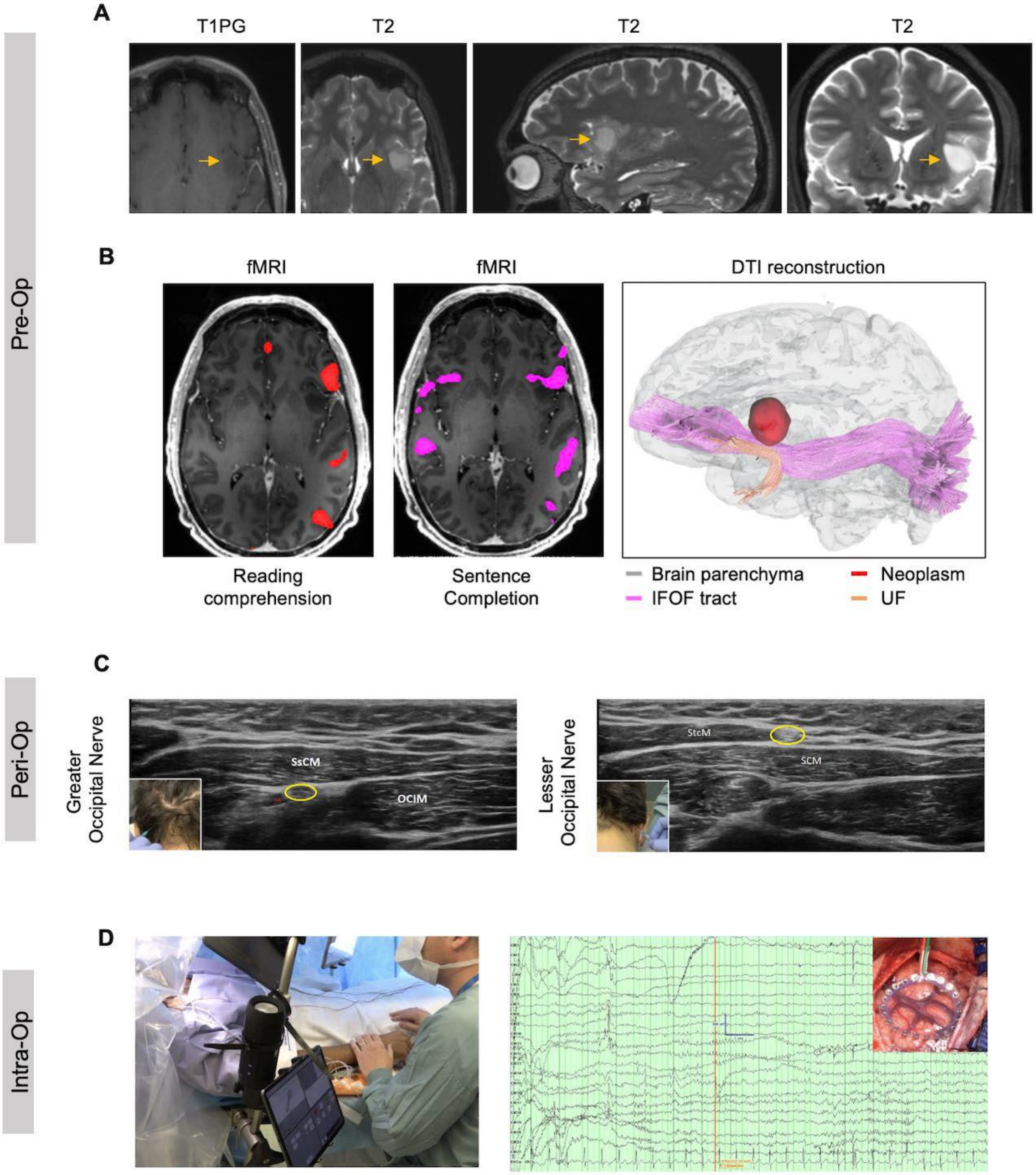

© 2024 Moniz-Garcia D et al. *JAMA Network Open.*

eFigure 2. Trend Plots Illustrating Cost Changes Over Time for Both Prestandardization and Poststandardization Cohorts

A, total Costs; B, costs with OR C, costs with room and board; and D, costs with health care professionals.

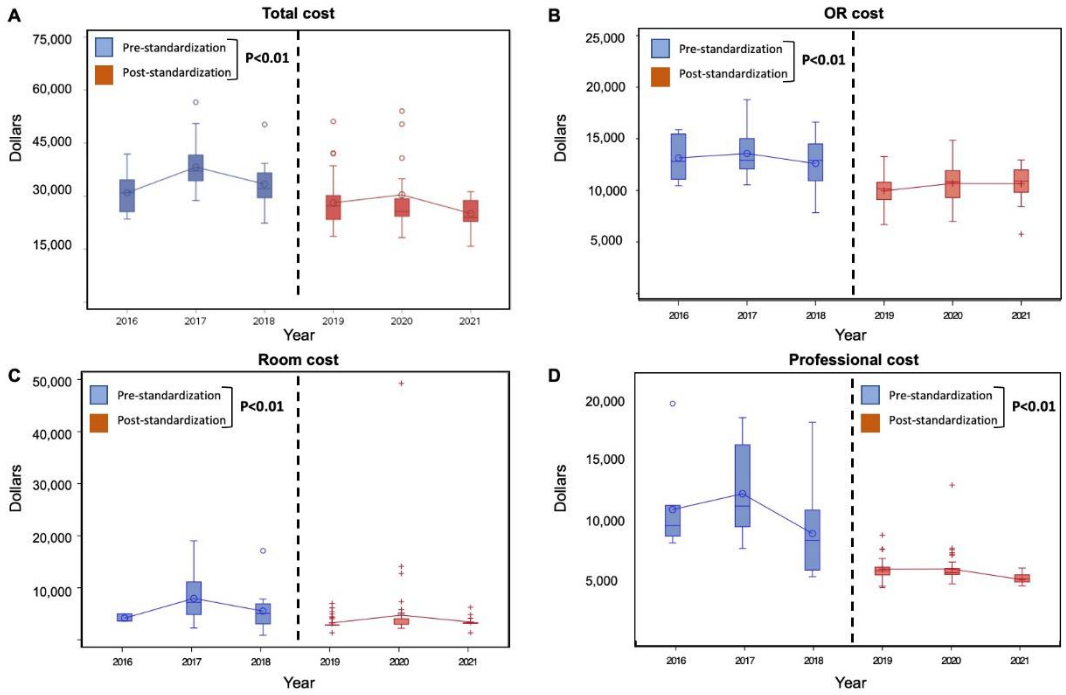

© 2024 Moniz-Garcia D et al. *JAMA Network Open.*

eTable. Sensitivity Analysis Considering Variable Inputs up to 1 SD

Cost categories ($)	Best case scenario			Worst case scenario		
	Pre-S	Post-S	Difference	Pre-S	Post-S	Difference
**Anesthesia costs**	35380.07	28513.98	-6866.09	36581.27	29397.04	-7184.23
**OR costs Pre-S**	33508.98	28955.51	-4553.47	38452.36	28955.51	-9496.85
**Other costs Pre-S**	32340.22	28955.51	-3384.71	39621.12	28955.51	-10665.61
**Professional costs Pre-S**	31519.30	28955.51	-2563.79	40442.04	28955.51	-11486.53
**Room costs Pre-S**	31939.92	28955.51	-2984.41	40021.42	28955.51	-11065.91
**OR costs Post-S**	35980.67	27147.36	-8833.31	35980.67	30763.66	-5217.01
**Other costs Post-S**	35980.67	20450.96	-15529.71	35980.67	37460.06	1479.39
**Professional costs Post-S**	35980.67	27751.8	-8228.87	35980.67	30159.22	-5821.45
**Room costs Post-S**	35980.67	24147.91	-11832.76	35980.67	33763.11	-2217.56

Pre-S: pre-standardization; Post-S: post-standardization

© 2024 Moniz-Garcia D et al. *JAMA Network Open.*
