# Supplementary material for: Awake Craniotomy Program Implementation
Source: JAMA Netw Open. 2024 Jan 24;7(1):e2352917. doi: 10.1001/jamanetworkopen.2023.52917 (PMC10809012; doi:10.1001/jamanetworkopen.2023.52917)
Supplement: Supplement 2. — Data Sharing Statement [file jamanetwopen-e2352917-s002.pdf]

Data Sharing Statement

Moniz-Garcia. Awake Craniotomy Program Implementation. *JAMA Netw Open*. Published January 24, 2024. doi:10.1001/jamanetworkopen.2023.52917

Data
Data available: Yes
Data types: Deidentified participant data
How to access data: garcia.diogo@mayo.edu; diogo.monizgarcia@gmail.com
When available: With publication

Supporting Documents
Document types: None

Additional Information
Who can access the data: Researchers requesting the data.
Types of analyses: For any purpose
Mechanisms of data availability: With signed data access agreement
